# Beyond the huddle: a comparative analysis of sense of belonging, group cohesion and interpersonal closeness in rugby, soccer, and basketball

**DOI:** 10.3389/fpsyg.2026.1878284

**Published:** 2026-09-22

**Authors:** Ivan Giuseppe Cammarata, Marina Galioto, Paula Tavares, Gabriele Zumbino, Antonino Bianco, Stefano Boca

**Affiliations:** 1 Department of Psychology, Educational Sciences and Human Movement, University of Palermo, Palermo, Italy; 2 Universidade de Coimbra Faculdade de Ciencias do Desporto e Educacao Fisica, Coimbra, Portugal

**Keywords:** group cohesion, interpersonal closeness, sense of belonging, social identity theory, sport contact level

## Abstract

This study investigates the psychosocial dynamics of team sports by comparing levels of sense of belonging, group cohesion, and perceived interpersonal closeness among athletes playing rugby, soccer, and basketball. Guided by Social Identity Theory, the research explored how the structural characteristics of each sport influence group bonds and individual feelings of inclusion. We hypothesized that the highly interdependent nature of rugby would lead to stronger group-level and individual-level connections than those found in soccer and basketball. Additionally, we posited that both cohesion and closeness would serve as significant predictors of an athlete’s sense of belonging, regardless of their sport. The sample included 169 adult athletes. The findings confirmed our hypotheses, revealing that rugby players reported the highest scores across all three measures. They showed a significantly higher sense of belonging, group cohesion and perceived interpersonal closeness than their soccer and basketball counterparts. The ANCOVA results further demonstrated that both group cohesion and perceived interpersonal closeness are strong positive predictors of an individual’s sense of belonging across all disciplines. This research highlights the profound impact a sport’s environment has on shaping the psychosocial experiences of its athletes. It confirms that while group cohesion and perceived interpersonal closeness are crucial for fostering a sense of belonging, the effectiveness of these bonds is amplified in sports that demand a high degree of physical and social interdependence. These insights offer valuable guidance for coaches and practitioners, suggesting that sport-specific strategies are necessary to enhance team functioning and athlete well-being.

## Introduction

In recent times, a considerable body of research has emerged in the field of psychology, with a focus on understanding the psychological factors that can influence athletes’ experiences, including confidence and self-efficacy (Martín-Rodríguez et al., 2024), motivation (Peng et al., 2025), mental toughness (Andersson et al., 2025), social support (Walton et al., 2024) and so on (eg., Vysochina and Vorobiova, 2019; Elbe and Wikman, 2017; Chang et al., 2020). While team identification, the cognitive and emotional significance of group membership, has been extensively studied in sport psychology (e.g., Bruner et al., 2018; Rees et al., 2015), the subjective experience of Sense of Belonging (SoB; Goodenow, 1993), remains underexplored, particularly in comparative designs across sports with varying interdependence. SoB not only predicts individual well-being and intrinsic motivation (Allen and Bowles, 2012) but also contributes to group-level outcomes such as collective efficacy and team resilience (Rees et al., 2015).

Following the Good’s definition of sense of belonging as “the feeling that one fits in, belongs to, or is a member of the… community in question” (Good et al., 2012, p. 700), we agree on its pivotal role for understanding athletes’ well-being, grounded on relational and motivational dynamics that sustain effective team functioning. Team sports naturally foster interactive and social contexts where identification with the group, mutual acceptance, and goals sharing create a supportive and motivating environment (Eys et al., 2019). Indeed, this sense of connection with others enhances well-being, strengthens motivation, and promotes both individual and collective performance, contributing to greater group cohesion (Stride et al., 2025).

Although group cohesion and sense of belonging are often considered together in sport psychology, they represent distinct but interrelated constructs. Group cohesion refers to the degree to which members of a group are united in the pursuit of common goals and in maintaining social bonds within the team (Carron and Brawley, 2012). It is typically conceptualized as a group-level construct with two main dimensions: task cohesion, reflecting the shared commitment to collective objectives, and social cohesion, capturing the quality of interpersonal relationships and the emotional bonds among members.

In contrast, sense of belonging is an individual-level psychological perception. It refers to the extent to which an individual feels accepted, valued, and connected within the group (Goodenow, 1993). While group cohesion emphasizes the structural and functional unity of the team, sense of belonging emphasizes the subjective experience of inclusion and acceptance from the perspective of each member. Since Sob is in relation with emotional patterns and cognitive processes, it follows that interpersonal closeness, or the perceived emotional bond between teammates, plays a central role in linking these constructs (Baumeister and Leary, 1995). Strong interpersonal connections enhance trust, cooperation, and mutual support, reinforcing group cohesion and individual sense of belonging. By fostering emotional attachment and positive social interactions, interpersonal closeness strengthens identification with the team and creates a motivating environment conducive to both personal well-being and collective performance (Aron et al., 1997).

Understanding the distinction between these constructs is critical, as they yield different outcomes. Group cohesion is consistently associated with enhanced team performance, persistence, and collective motivation (Carron et al., 2002a), whereas a sense of belonging is more strongly linked to individual well-being, self-esteem, and intrinsic motivation (Allen and Bowles, 2012). Thus, integrating both perspectives provides a more comprehensive framework for examining athletes’ psychosocial experiences within team environments. The present study aims to examine how group cohesion, interpersonal closeness and sense of belonging vary across athletes participating in three distinct team sports: soccer, basketball, and rugby. By investigating these differences, the current research seeks to contribute to a more nuanced understanding of the interplay between group-level and individual-level psychosocial factors in sport, providing insights relevant for researchers and practitioners aiming to enhance team functioning and athlete well-being.

## Social identity theory

The Social Identity Theory (SIT) (Turner et al., 1979), later expanded through the Self-Categorization Theory (Turner et al., 1987), offers a robust framework to understand how individuals derive meaning and motivation from their membership in social groups.

According to SIT, the self-concept is not solely defined by personal identity but also by social identity, which represents the component of the self-derived from group memberships that are perceived as meaningful. Within this perspective, three interrelated psychological processes are central to the development and maintenance of social identity. First, through social categorization, individuals cognitively organize their social environment by classifying themselves and others into distinct groups, such as “our team” versus “the opponent,” thereby creating the foundation for group-based dynamics. Second, social identification occurs when individuals internalize their group membership as part of their self-concept, developing a sense of emotional connection, loyalty, and commitment to the group’s values and objectives. Finally, social comparison involves evaluating the ingroup in relation to relevant outgroups, often favoring the former to maintain or enhance collective and individual self-esteem.

In sport contexts, where collective goals and interdependence are crucial, these processes are particularly salient. Social categorization enhances a shared team identity, social identification strengthens athletes’ motivation and adherence to group norms, and social comparison reinforces pride and purpose, particularly in competitive situations (Tajfel and Turner, 1979; Rees et al., 2015).

Since individuals required to be in a group challenging another group with the purpose to win can showcase disloyalty and aggressiveness toward competitors and more closeness to relative group members (Fine, 2004), investigating intergroup dynamics within sport teams can contribute to highlight significant patterns in psychology applied to sport research.

To explore these dynamics, this study analyzes differences across rugby, soccer, and basketball, as each sport embodies a distinct level of interdependence and social unity. In rugby, interdependence is continuous and physical. Actions like the scrum or maul require perfect coordination and a collective physical effort, creating a unique experience of group identity. This environment strongly reinforces social categorization (“us” vs. “them”) and social identification because success is entirely dependent on group unity. As noted by Haslam et al. (2011) in their work on social identity and well-being, such contexts are ripe for fostering a strong “we-ness.”

In soccer, while interdependence is crucial, there are also moments of more individual performance. A single player’s creative skill, a dribble, or a decisive pass can change the outcome of a game, making the link between individual and collective performance more variable. While a meta-analysis by Carron et al. (2002b) confirmed that cohesion is a robust predictor of success in soccer, the nature of the game may not foster a sense of collective belonging in the same continuous manner as rugby.

Finally, in basketball, the fast-paced nature and reliance on quick transitions create a dynamic interdependence. The game requires high-speed collaboration and a constant reading of teammates’ intentions, which can build a form of cohesion based on reciprocal trust and quick decision-making (Vysochina and Vorobiova, 2019). However, the structure of the game, with less direct physical contact and a greater emphasis on the performance of a few key players at crucial moments, may shape both the sense of belonging and group cohesion in a way that is distinct from the sustained, physical nature of rugby.

The present study aims to examine how group cohesion and sense of belonging vary across athletes participating in these three team sports.

Based on this theoretical framework, the study aims to test the following hypotheses:

*H1*: Group cohesion will be significantly higher among rugby athletes compared to athletes in soccer and basketball.

*H2*: Sense of belonging will be significantly higher among rugby athletes compared to athletes in soccer and basketball.

*H3*: Perceived interpersonal closeness will be significantly higher among rugby players compared to soccer and basketball players.

*H4*: Group cohesion and interpersonal closeness will have a significant positive influence on sense of belonging across all sport disciplines.

## Methods

### Participants, design and procedure

The present study employed a rigorous, multi-phase methodological approach aimed at ensuring data collection and analysis were fully aligned with the formulated hypotheses. The target population consisted of adult athletes actively participating in team sports, and three distinct groups were selected to represent specific disciplines: soccer, basketball, and rugby.

Participant recruitment was carried out using a mixed-method strategy designed to engage a sufficient number of athletes from competitive sports contexts. In the initial phase, some participants were contacted directly via online channels, including social networks, email, and instant messaging applications. These athletes were provided with a link to the digital questionnaire, accompanied by a concise introduction to the study, detailed information on data protection procedures, and an informed consent form.

To further broaden the sample and include athletes embedded within team contexts, direct contact was established with various sports clubs. In certain instances, teams welcomed the participation request and invited the researcher to attend one or more training sessions, allowing for a direct presentation of the study to the players, who then completed the digital questionnaire on-site. In other cases, contact with clubs occurred via telephone, during which the study was introduced, and formal consent was requested from coaches or club managers. Only upon explicit approval from these authorities was data collection permitted within the team setting.

Once participants were identified, the psychometric instrument was administered individually in digital format. Prior to completion, participants were informed of the study’s objectives, procedures, and guarantees of anonymity, and were reminded of their right to withdraw at any time without consequence. A brief oral introduction was provided to ensure understanding of the materials and proper completion of the questionnaire, while fostering a climate of trust and collaboration.

This comprehensive and flexible recruitment and administration approach enabled the inclusion of a diverse sample in terms of age, sport discipline, and level of participation, thereby enhancing representativeness relative to the target population and providing a robust foundation for subsequent data analysis. Data collection was conducted entirely online, and the study received ethical approval from University of Palermo (no 68176-2026) prior to its commencement.

The total sample consisted of 169 participants. The rugby group included 51 athletes (M_age_ = 25.5, SD = 3.64; M_years of practice_ = 2.9), the soccer group comprised 66 athletes (M_age_ = 24.3, SD = 3.7; M_years of practice_ = 3), and the basketball group included 52 athletes (M_age_ = 24, SD = 3.4; M_years of practice_ = 2.7). Overall, the participants had a mean age of 24.5 years (SD = 3.6) and an average of 8.14 years of sports experience. Regarding the competitive level, all athletes recruited for this study competed at a semi-professional level within their respective regional sports leagues. This homogeneity ensures that the differences observed among the groups are not confounded by disparities between recreational and elite athletic environments.

### Measures

#### Sense of belonging

Participants’ sense of belonging was assessed using the sense of belonging Scale (SoBS; Goodenow, 1993). The instrument demonstrated excellent internal consistency, with a Cronbach’s alpha of 0.96, indicating high reliability in this sample. The scale comprises 12 items organized into two subscales: Social Acceptance (*α* = 0.92) and Sense of Inclusion (*α* = 0.94). Responses were provided on a 5-point Likert scale ranging from 1 (strongly disagree) to 5 (strongly agree).

#### Cohesion

Group cohesion was assessed using the Group Environment Questionnaire (GEQ; Carron, 1985), adapted by the authors (see Results). The scale demonstrated excellent reliability in the present sample (*α* = 0.97) and comprises 16 items across four subscales: Social Cohesion (*α* = 0.92), Task Cohesion (*α* = 0.94), Social Attraction to the Group (*α* = 0.96), and Task Attraction to the Group (*α* = 0.92). Responses were recorded on a 9-point Likert scale ranging from 1 to 9.

#### Interpersonal closeness

Perceived interpersonal closeness was assessed using the Inclusion of Other in the Self Scale (IOS; Aron et al., 1992). The IOS is a single-item graphic scale commonly used in social psychology to measure the degree to which one perceives another as part of the self. It consists of seven pairs of concentric circles representing increasing levels of inclusion, from complete separation (1) to almost complete overlap (7). Participants were asked to select the pair of circles that best reflected their perceived closeness to other team members. Responses were scored from 1 to 7, with higher scores indicating greater perceived inclusion. Although the single-item nature of the IOS scale precludes the calculation of internal consistency coefficients (e.g., Cronbach’s alpha), its selection over multi-item alternatives is theoretically and empirically grounded. Aron et al. (1992) demonstrated that the IOS possesses strong psychometric properties, including excellent test–retest reliability and high concurrent validity with complex, multi-item measures of intimacy and interpersonal connectedness. Furthermore, its pictorial format effectively captures the holistic, subjective perception of interpersonal bonds while minimizing cognitive load and survey fatigue. Therefore, the IOS scale is considered an efficient and robust primary measure for assessing the subjective emotional bonds within these team sport contexts.

### Statistical analysis

Analyses were conducted using the RStudio graphical interface (Posit team, 2025) with the psych (Revelle and Revelle, 2015) and lavaan (Rosseel, 2012) packages. First, descriptive statistics (mean, standard deviation, skewness, and kurtosis) were calculated for all items of the sense of belonging Scale and Group Environment Questionnaire. To assess univariate normality, skewness and kurtosis thresholds of ±2.00 and ±7.00, respectively, were adopted (Curran et al., 1996). Items exceeding these criteria were excluded from subsequent analyses.

Second, Confirmatory Factor Analyses (CFA) were conducted on both scales using the Diagonally Weighted Least Squares (DWLS) estimator (Brown, 2015). Model fit was assessed through the Chi-square test (χ^2^) and the RMSEA index with 90% confidence interval, with values below 0.05 indicating good fit and values between 0.05 and 0.08 considered acceptable. Additional indices included the Comparative Fit Index (CFI) and the Tucker–Lewis Index (TLI), where values above 0.95 indicated good fit and values above 0.90 acceptable fit (Kline, 2015). Factor loadings (λ) greater than 0.50 were retained (Brown, 2015).

After confirming the psychometric properties of the instruments, differences between sport groups (soccer, basketball, and rugby) in relation to sense of belonging, group cohesion and perceived interpersonal closeness were examined through one-way Analyses of Variance (ANOVAs). To control for the family-wise Type I error rate arising from testing nine dependent variables, a Bonferroni correction was applied to the alpha level for the omnibus ANOVA F-tests. For each ANOVA that yielded a significant omnibus effect, pairwise group comparisons were subsequently examined using Tukey’s Honestly Significant Difference (HSD) test, which controls the family-wise error rate within each set of comparisons via the studentized range distribution and therefore did not require additional Bonferroni adjustment. An *a priori* power analysis for a one-way Analysis of Variance (ANOVA) was conducted using the pwr package in R (Champely, 2020). Assuming three groups (*k* = 3), a medium effect size (*f* = 0.25; Cohen, 1988), an alpha level of 0.05, and desired power of 0.80, the analysis indicated that approximately 52 participants per group were required. The final sample included 169 participants, exceeding the minimum required to achieve adequate statistical power. When significant effects emerged, Tukey *post-hoc* tests were performed to identify pairwise differences. In addition, an Analysis of Covariance (ANCOVA) was conducted to investigate whether overall group cohesion and perceived interpersonal closeness influenced levels of sense of belonging across the different sport disciplines. However, it should be noted that while the soccer and basketball groups met or exceeded this threshold, the rugby subsample comprised 51 participants, falling marginally below the requirement by a single participant. Given the negligible nature of this deficit (*n* = 1) and the robust overall sample size (*N* = 169), statistical power was not meaningfully compromised, and the analyses remained adequately powered to detect the anticipated effects.

## Results

### Descriptive statistics and confirmatory factor analyses

#### Sense of belonging scale

All item of the SoBs exhibited skewness and kurtosis within the thresholds (see Appendix Table A1). A confirmatory factor analysis (CFA) on the 12 items was conducted to evaluate the measurement structure. All standardized factor loadings were above λ > 0.8, with values ranging from 0.846 to 0.960 for the “Social Acceptance” factor and from 0.882 to 0.955 for the “Sense of Inclusion” factor (see Figure 1; Appendix Table A2), indicating that a large portion of item variance is explained by the specified factors. The two-factor model demonstrated an overall good fit (see Table 1). Although the Chi-square test was significant, χ^2^(53, *N* = 169) = 102.47, *p* < 0.001, this result is likely influenced by the high sensitivity of this statistic to sample size (Babyak and Green, 2010). Therefore, greater emphasis was placed on alternative fit indices. The Comparative Fit Index (CFI = 0.998) and Tucker-Lewis Index (TLI = 0.998) suggested excellent fit, while the Root Mean Square Error of Approximation (RMSEA = 0.075, 90% CI = 0.053–0.096) was within an acceptable range, indicating a reasonable approximation of the observed covariance structure. Additionally, the Standardized Root Mean Square Residual (SRMR = 0.043) further supported good model fit.

**Figure 1:**
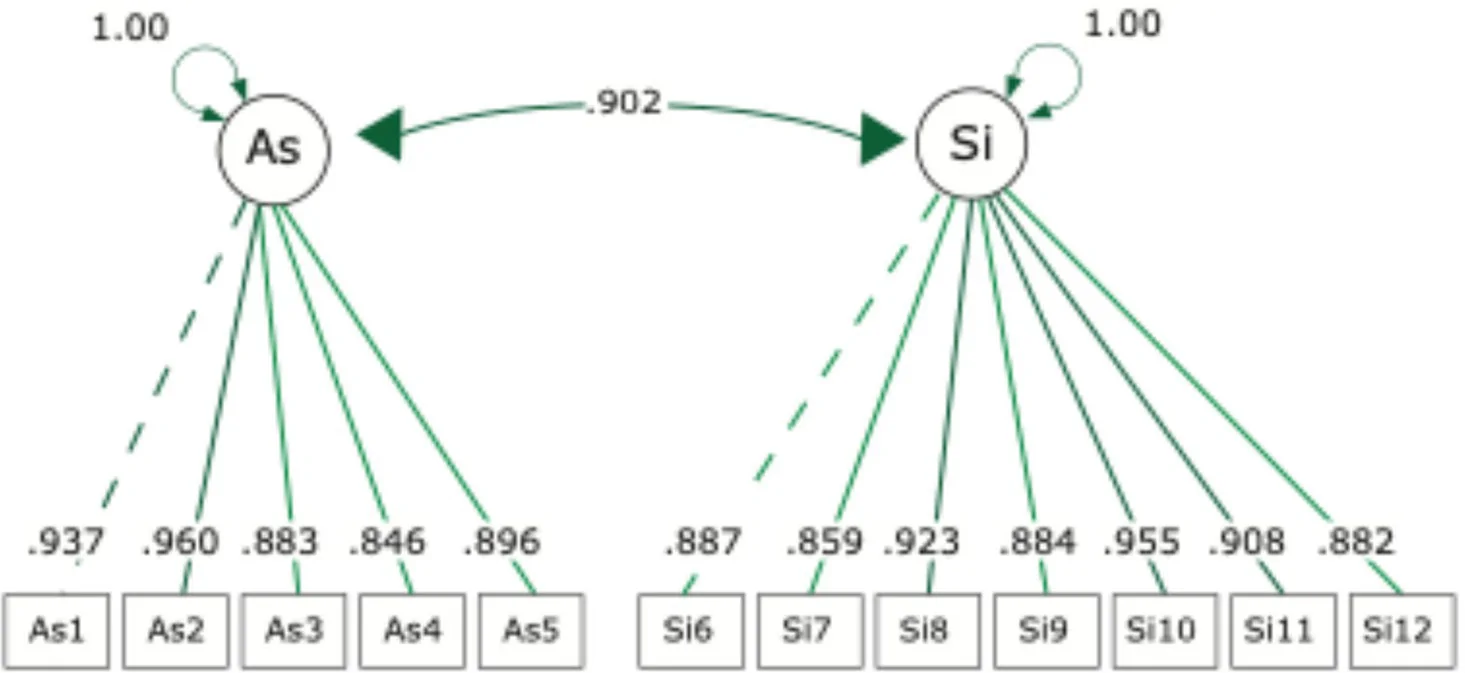
Factor solution diagram. As, Social Acceptance; Si, Sense of Inclusion.

**Table 1:** Goodness-of-fit indicators for CFA.

Model	χ^2^	df	*p*-value (χ^2^)	CFI	TLI	RMSEA	SRMR
Two-factors SoBs	102.47	169	<0.001	0.998	0.998	0.075	0.043
Four-factors GEQ	180.87	169	<0.001	0.984	0.981	0.071	0.041

#### Group environment questionnaire

To ensure the validity of the adapted Group Environment Questionnaire (Carron et al., 1985), a systematic adaptation process was conducted. First, the original instrument was translated into Italian following a forward-backward translation protocol by two independent bilingual experts. Discrepancies were resolved through discussion to ensure semantic equivalence. Subsequently, a pilot test was conducted with a small sample of 25 participants to verify item clarity and comprehension, resulting in no substantial modifications.

All item of the GEQ exhibited skewness and kurtosis within the thresholds (see Appendix Table A3). To verify the adequacy of the adapted version’s factor structure, a Confirmatory Factor Analysis (CFA) was conducted on a four-factor model. Standardized factor loadings were strong across all items, ranging from 0.818 to 0.956, indicating that each item substantially contributes to its respective latent factor (see Figure 2; Appendix Table A4).

**Figure 2:**
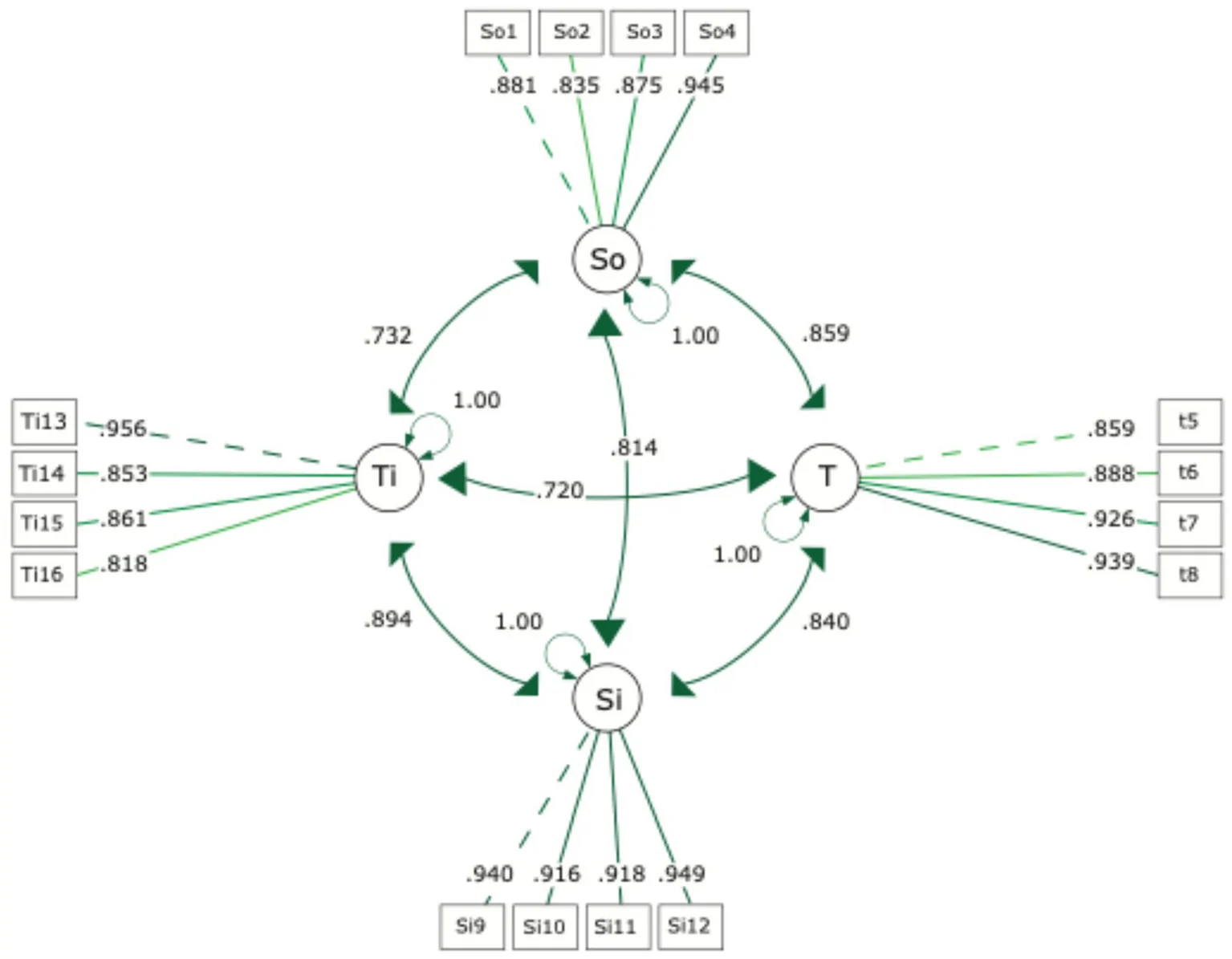
Factor solution diagram. So, social cohesion; T, task cohesion; Si, social attraction to the group; Ti, task attraction to the group.

The model demonstrated an overall good fit. Although the Chi-square test was significant, χ^2^(98, *N* = 169) = 180.87, *p* < 0.001, this is expected given the sensitivity of the Chi-square statistic to sample size. Alternative fit indices suggested satisfactory to excellent model fit (see Table 1). The Comparative Fit Index (CFI = 0.984) and Tucker-Lewis Index (TLI = 0.981) indicated excellent fit, while the Root Mean Square Error of Approximation (RMSEA = 0.071, 90% CI = 0.055–0.087) was within an acceptable range. The Standardized Root Mean Square Residual (SRMR = 0.041) also supported good model fit.

All latent factors were significantly correlated, with standardized covariances ranging from 0.720 to 0.894, suggesting that while factors are related, they capture distinct dimensions. Item residual variances were all positive and within acceptable ranges, further supporting the adequacy of the measurement model. Overall, the results indicate that the four-factor structure provides a robust representation of the data, with each factor well-defined by its indicators and the overall model fitting the observed covariance structure satisfactorily.

### Analyses of variance

One-way ANOVAs were conducted to examine differences across sport groups (soccer, basketball, and rugby) in sense of belonging, group cohesion, and perceived interpersonal closeness.

Sense of belonging differed significantly across groups, *F*(2, 166) = 13.74, *p* < 0.001, η^2^ = 0.14. *Post-hoc* tests revealed that rugby players reported the highest sense of belonging, followed by soccer players, while basketball players scored lowest. Specifically, soccer exceeded basketball (diff = 5.17, *p* = 0.003), rugby exceeded basketball (diff = 8.62, *p* < 0.001), and rugby was marginally higher than soccer (diff = 3.45, *p* = 0.074).

Subscale social acceptance differed significantly across groups, *F*(2, 166) = 10.31, *p* < 0.001, η^2^ = 0.11. *Post-hoc* Tukey’s HSD indicated soccer > basketball (diff = 1.80, *p* = 0.016), rugby > basketball (diff = 3.08, *p* < 0.001), and no significant difference between rugby and soccer (diff = 1.29, *p* = 0.118).

Subscale sense of inclusion showed significant differences, *F*(2, 166) = 14.28, *p* < 0.001, η^2^ = 0.15, with the strongest effects. *Post-hoc* tests confirmed soccer > basketball (diff = 3.37, *p* = 0.002), rugby > basketball (diff = 5.54, *p* < 0.001), and rugby marginally higher than soccer (diff = 2.17, *p* = 0.076) (see Figure 3).

**Figure 3:**
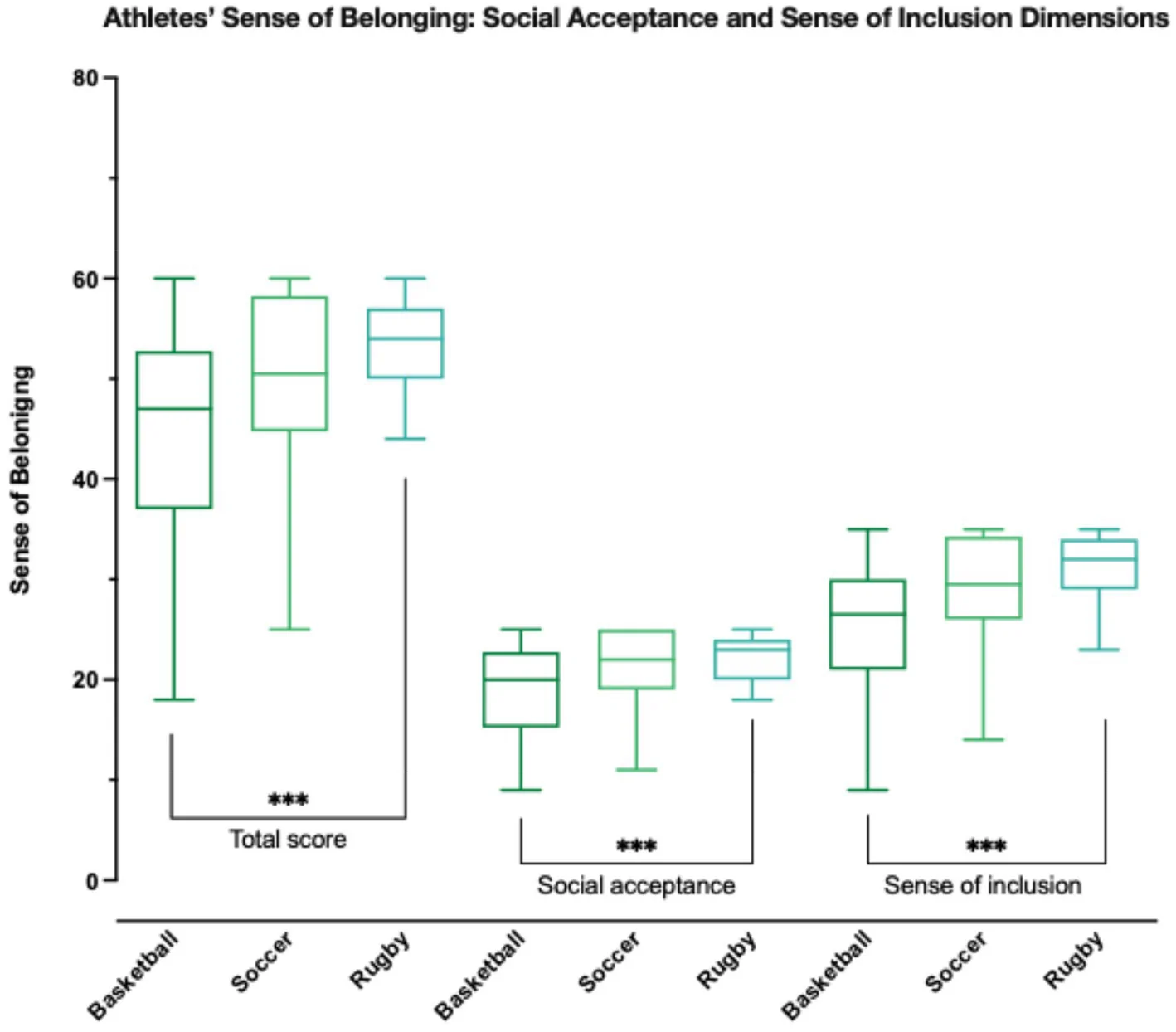
Sense of belonging and its subscales across groups. ***p < 0.001.

A significant group effect emerged for overall group cohesion, *F*(2, 166) = 17.65, *p* < 0.001. Rugby teams reported the highest cohesion scores, followed by soccer, with basketball teams showing the lowest levels. *Post hoc* comparisons confirmed that rugby teams scored significantly higher than both soccer (diff = 13.60, *p* < 0.001) and basketball teams (diff = 23.03, *p* < 0.001), and soccer teams scored higher than basketball teams (diff = 9.44, *p* = 0.029) (see Figures 4, 5).

**Figure 4:**
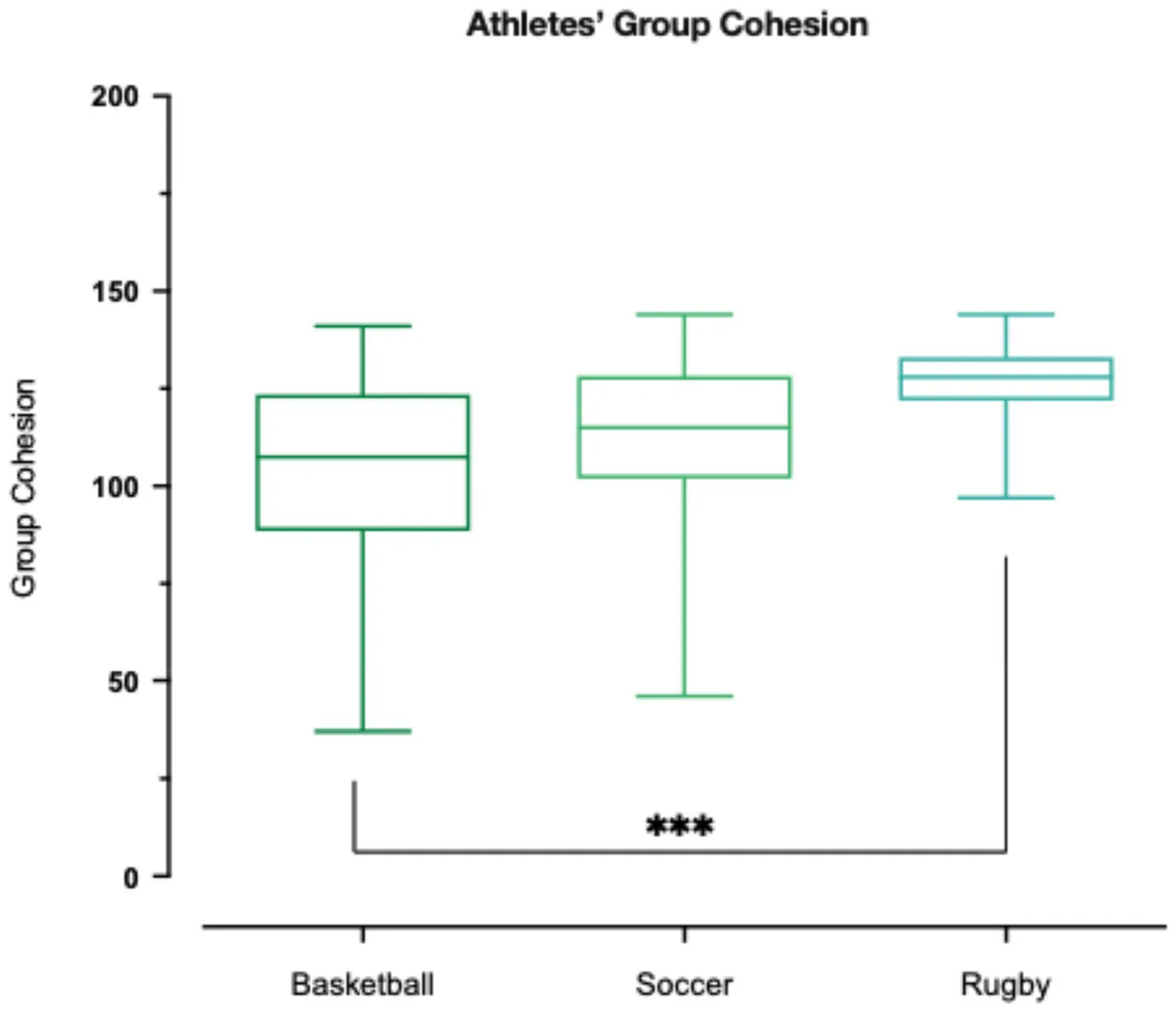
Total group cohesion: comparative scores by sport. ***p < 0.001.

**Figure 5:**
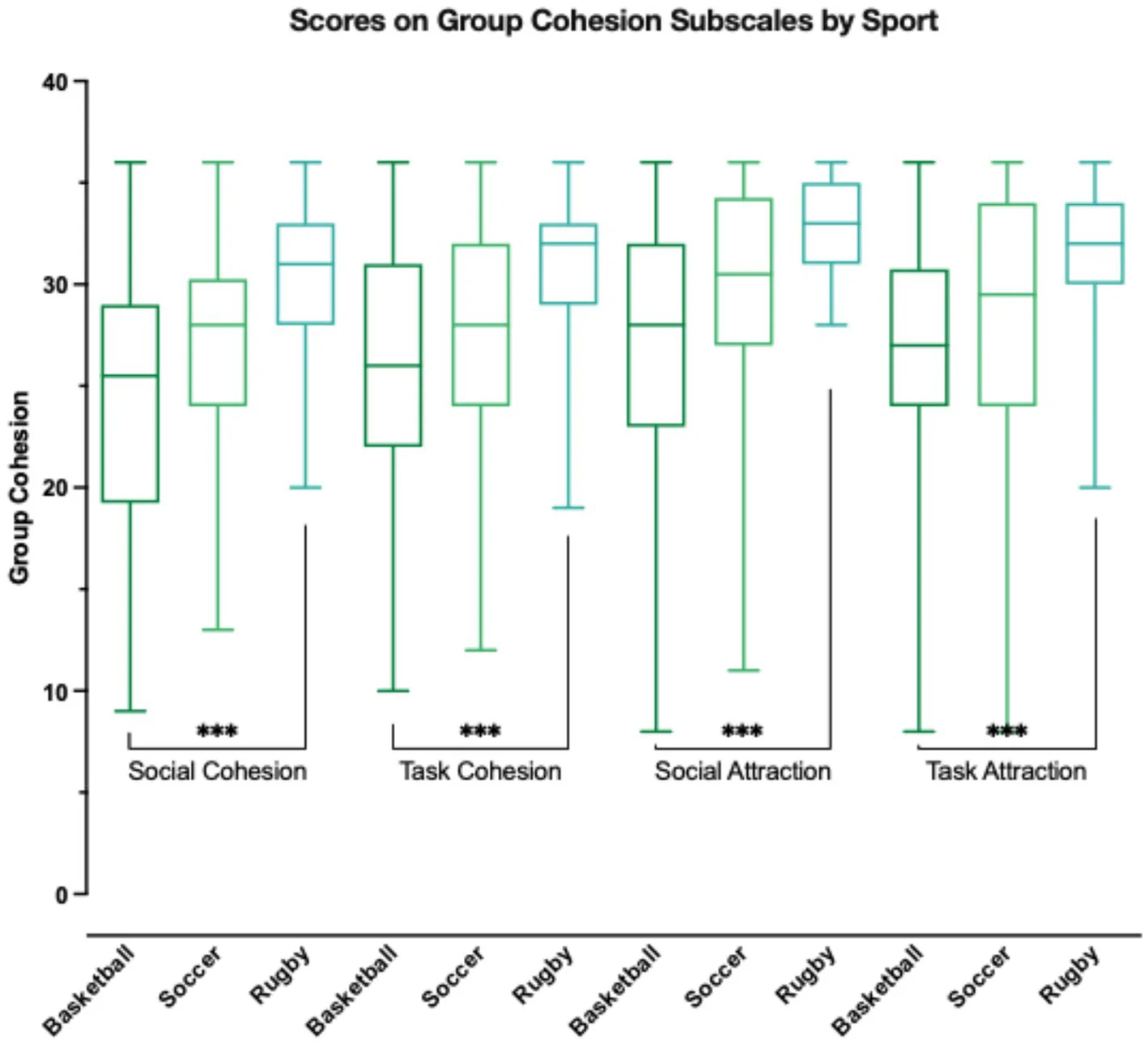
Comparison of task and social subscale scores among athletes from different sports. ***p < 0.001.

For the social cohesion subscale, a significant group effect was found, *F*(2, 166) = 15.94, *p* < 0.001, η^2^ = 0.16. Tukey’s HSD tests indicated that rugby teams scored higher than soccer teams (diff = 3.41, *p* = 0.002), soccer teams scored higher than basketball teams (diff = 2.55, *p* = 0.030), and rugby teams scored higher than basketball teams (diff = 5.97, *p* < 0.001).

For the task cohesion subscale, the group effect was also significant, *F*(2, 166) = 12.47, *p* < 0.001, η^2^ = 0.13. *Post hoc* analyses revealed that rugby teams scored higher than both soccer (diff = 3.33, *p* = 0.003) and basketball teams (diff = 5.19, *p* < 0.001), whereas the difference between soccer and basketball teams was not significant (diff = 1.86, *p* = 0.148).

For the social integration subscale, a significant group effect emerged, *F*(2, 166) = 15.94, *p* < 0.001, η^2^ = 0.16. Tukey’s HSD indicated that rugby teams scored higher than soccer (diff = 3.30, *p* = 0.006) and basketball teams (diff = 6.27, *p* < 0.001), and that soccer teams scored higher than basketball teams (diff = 2.97, *p* = 0.014).

Finally, for the task integration subscale, a significant group effect was observed, *F*(2, 166) = 11.50, *p* < 0.001, η^2^ = 0.12. Tukey’s HSD revealed that rugby teams scored higher than soccer (diff = 3.56, *p* = 0.005) and basketball teams (diff = 5.61, *p* < 0.001), whereas soccer and basketball teams did not differ significantly (diff = 2.05, *p* = 0.160).

Perceived interpersonal closeness also varied by sport, *F*(2, 166) = 7.52, *p* = 0.001, η^2^ = 0.08. Rugby players reported greater closeness than both soccer (diff = 0.64, *p* = 0.032) and basketball players (diff = 1.02, *p* < 0.001), while soccer and basketball did not differ significantly (diff = 0.38, *p* = 0.28).

Overall, these results indicate that rugby teams show the strongest group connectedness across all measures, soccer teams are intermediate, and basketball teams consistently report the lowest levels.

### Analyses of covariance

An ANCOVA was conducted to examine differences in sense of belonging across sport groups (soccer, basketball, and rugby), while controlling for overall group cohesion and perceived interpersonal closeness. Results indicated significant main effects of sport group, *F*(2, 160) = 36.38, *p* < 0.001, overall group cohesion, *F*(1, 160) = 257.10, *p* < 0.001, and interpersonal closeness, *F*(1, 160) = 12.90, *p* < 0.001, demonstrating that sense of belonging varies by sport and is strongly influenced by both cohesion and closeness. The interaction between sport group and group cohesion was marginally significant, *F*(2, 160) = 2.62, *p* = 0.076, suggesting a tendency for the effect of cohesion on sense of belonging to differ slightly across sports, with cohesion potentially having a stronger impact in some groups (e.g., rugby) than others (e.g., basketball and soccer) (see Figure 6). The interaction between sport group and interpersonal closeness was not significant, *F*(2, 160) = 2.13, *p* = 0.122, indicating that the effect of perceived closeness on sense of belonging is consistent across sports. These findings highlight the central role of group cohesion in fostering a sense of belonging, while also suggesting that sport-specific dynamics may moderate this relationship to a limited extent.

**Figure 6:**
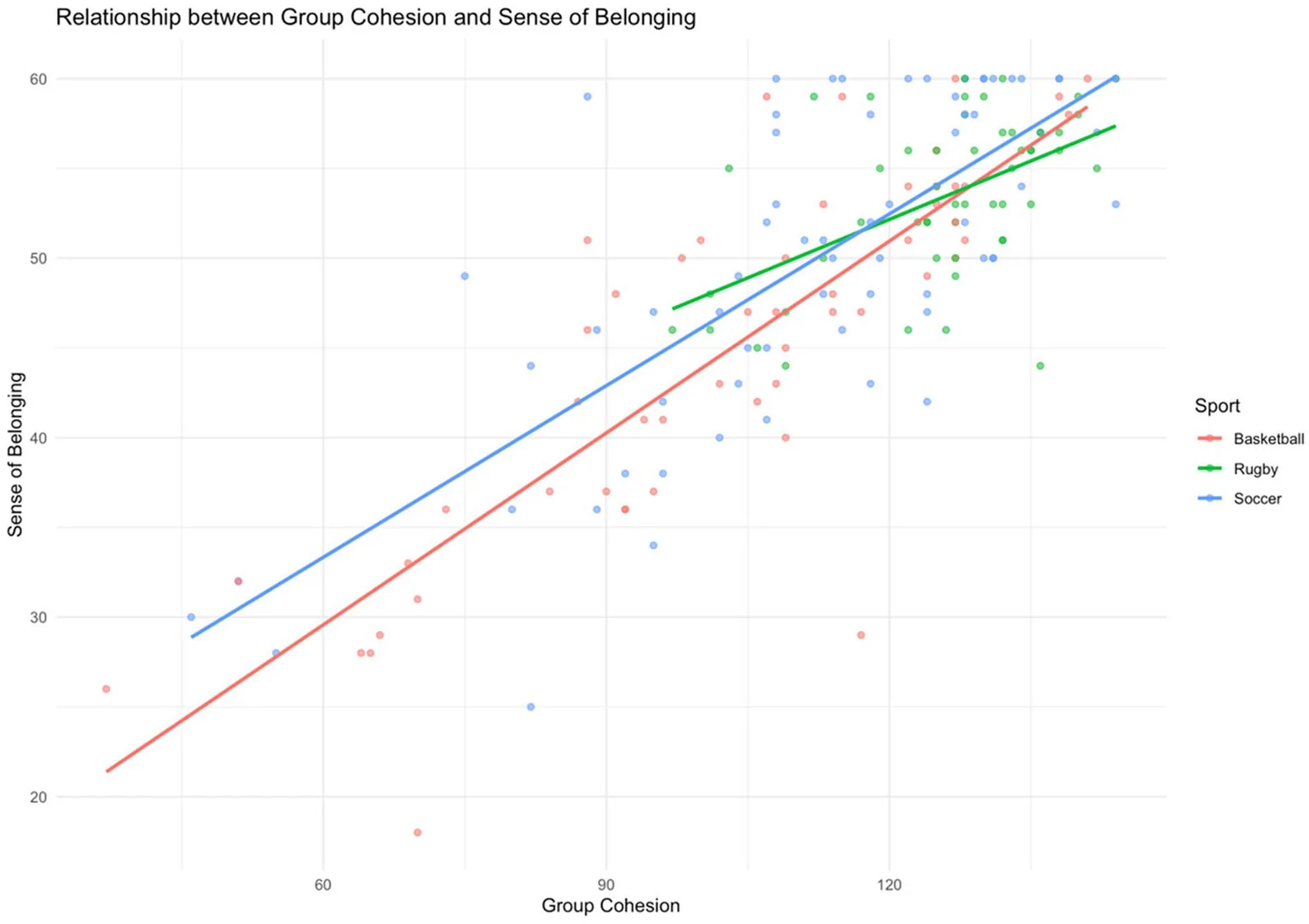
Relationship between group cohesion and sense of belonging across sport groups.

## Discussion

The findings of this study offer a nuanced understanding of psychosocial dynamics within team sports, particularly the distinct roles of group cohesion, interpersonal closeness, and a sense of belonging. The results confirmed our hypotheses, indicating that rugby players reported the highest levels of group cohesion, a sense of belonging, and perceived interpersonal closeness, with soccer players in an intermediate position and basketball players scoring the lowest across all three measures. These findings are consistent with our theoretical framework, suggesting that the continuous, highly interdependent nature of rugby, which necessitates shared physical effort and collective goal pursuit, fosters a stronger social identity among its members. This environment seems to translate into a powerful “we-ness,” reinforcing both the group-level unity and the individual’s psychological experience of acceptance and inclusion. The data also confirmed that both group cohesion and perceived interpersonal closeness are significant predictors of a sense of belonging, highlighting their central role in an athlete’s well-being and in fostering positive group dynamics across all sports. While a marginal interaction effect between sport and cohesion was observed, the consistent influence of cohesion on belonging underscores its universal importance.

The study aligns with the Social Identity Theory (SIT), suggesting an association between the structural characteristics of a sport and the formation and maintenance of a strong social identity. The high interdependence and collective physical effort inherent in rugby are associated with greater group cohesion and a more pronounced sense of belonging compared to sports with different dynamics, such as soccer and basketball. However, it is crucial to avoid drawing strictly causal conclusions. While the sporting environment may actively shape group dynamics, alternative explanations for the observed differences must be considered. First, a self-selection bias may exist, wherein pre-existing personality traits, such as trait agreeableness, extraversion, or a natural disposition toward teamwork, lead certain individuals to gravitate toward highly cohesive sports like rugby. Second, the observed variations could be significantly influenced by distinct cultural norms, traditions, and socialization processes embedded within each sport’s broader community, rather than solely by the physical structure and rules of the game itself.

These dynamics are consistent with recent evidence demonstrating the broader psychosocial impact of athletic involvement. For instance, Galioto et al. (2025) found that university students engaged in sport and exercise programs exhibit a much stronger sense of institutional belonging than non-sport students, largely because the habit of practicing sports, training together, and pursuing team achievements naturally enhances social connectedness and mutual support.

Furthermore, the findings of this study can be fruitfully interpreted through the lens of Social Capital Theory. The core variables examined, sense of belonging, group cohesion, and interpersonal closeness, act as vital “thermometers” or manifestations of a group’s social capital. In team sports, the repeated interactions, shared collective goals, and mutual trust required for success inherently build robust relational networks. By fostering these strong psychosocial bonds, highly interdependent sports environments, such as rugby, do not merely enhance athletic performance; they also serve as critical engines for broader social development. When athletes experience high levels of cohesion, intimacy, and belonging within their teams, they develop cooperative skills, reciprocity, and civic trust that often extend beyond the sporting context, ultimately contributing to the social capital of their wider communities.

The findings offer concrete guidance for coaches and sport practitioners, highlighting that a one-size-fits-all approach is insufficient and that psychological management of a team should consider the specific characteristics of each discipline. In rugby, where group cohesion and sense of belonging are high, coaches can focus on maintaining and reinforcing these bonds, leveraging the inherent interactions of the game to further solidify team unity. In soccer and basketball, where group cohesion and sense of belonging are less pronounced, it is important to proactively implement strategies that foster team building, promote shared objectives, and cultivate interpersonal closeness, creating an environment in which athletes feel free to connect on a personal level.

In conclusion, group cohesion and perceived interpersonal closeness are essential for fostering a strong sense of belonging, but maximizing these outcomes requires sport-specific approaches that align with the unique dynamics of each team, ultimately enhancing both athlete well-being and performance.

## Conclusion

This study provides novel insights into the psychosocial dynamics of team sports by examining how group cohesion and sense of belonging vary across athletes in soccer, basketball, and rugby. The findings highlight the importance of sport-specific structural characteristics, demonstrating that high interdependence and collective effort, as seen in rugby, foster stronger group cohesion and a more pronounced sense of belonging. Moreover, perceived interpersonal closeness emerged as a key predictor of individual belonging across all sports, emphasizing the central role of social bonds in shaping athletes’ experiences and well-being.

Some limitations should be acknowledged. First, the cross-sectional design precludes causal inferences regarding the relationship between sport type, group cohesion, and sense of belonging. Consequently, we cannot confidently assert that participating in a highly interdependent sport causes higher levels of belongingness. The reverse directionality must be considered as a potential confounding factor: a self-selection bias may be present, meaning that individuals with a stronger dispositional preference for close interpersonal bonds and teamwork may intentionally self-select into sports characterized by greater interdependence. Second, the study relied on self-report measures, which may be subject to social desirability and response biases.

Future research should address these limitations by employing longitudinal designs to investigate the development of group cohesion and sense of belonging over time and across competitive seasons. Expanding the range of sports, including individual and mixed-team contexts, would enhance generalizability and deepen understanding of the mechanisms driving social identity and interpersonal closeness within membership. Additionally, incorporating observational or behavioral measures could complement self-reports and provide a more comprehensive view of team dynamics. Overall, such research will further inform interventions aimed at optimizing both athlete well-being and team performance across diverse sporting contexts.

## Data Availability

The original contributions presented in the study are included in the article/Supplementary material, further inquiries can be directed to the corresponding authors.
