# Supplementary material for: Beyond the huddle: a comparative analysis of sense of belonging, group cohesion and interpersonal closeness in rugby, soccer, and basketball
Source: Front Psychol. 2026 Sep 22;17:1878284. doi: 10.3389/fpsyg.2026.1878284 (PMC13638397; doi:10.3389/fpsyg.2026.1878284)
Supplement: Supplementary file 1 [file Supplementary_file_1.docx]

**Appendix**

Table A1. Descriptive of the original pool of 12 items of SoBs scale

| *Item* | *M* | *SD* | *Skew* | *Kurtosis* |
| --- | --- | --- | --- | --- |
| SBSas1 | 4.22 | 0.79 | -0.76 | 0.01 |
| SBSas2 | 4.31 | 0.79 | -0.90 | 0.05 |
| SBSas3 | 4.12 | 0.84 | -0.70 | -0.18 |
| SBSas4 | 4.08 | 0.93 | -1.00 | 0.88 |
| SBSas5 | 4.21 | 0.85 | -0.83 | -0.11 |
| SBSsi6 | 4.24 | 0.87 | -0.91 | -0.04 |
| SBSsi7 | 4.08 | 0.98 | -0.76 | -0.39 |
| SBSsi8 | 4.09 | 0.98 | -0.89 | 0.08 |
| SBSsi9 | 3.92 | 0.95 | -0.46 | -0.59 |
| SBSsi10 | 4.12 | 0.91 | -0.81 | 0.03 |
| SBSsi11 | 4.06 | 0.93 | -0.78 | -0.06 |
| SBSsi12 | 3.94 | 0.97 | -0.80 | 0.36 |

Note: SBS= sense of beloning; as= Social acceptance; si= sense of inclusion.

Table A2. Unstandardized Loadings (with standard errors) and Standardized Loadings of the entire pool of 12 items of SoBs scale included in the CFA.

| *Item* | *Unstandardized Loadings* | *Standardized Loadings* |
| --- | --- | --- |
| SBSas1 | 1.000 (-) | 0.937 |
| SBSas2 | 1.025 (0.022) | 0.960 |
| SBSas3 | 0.943 (0.027) | 0.883 |
| SBSas4 | 0.903 (0.032) | 0.846 |
| SBSas5 | 0.956 (0.027) | 0.896 |
| SBSsi6 | 1.000 (-) | 0.887 |
| SBSsi7 | 0.969 (0.036) | 0.859 |
| SBSsi8 | 1.040 (0.027) | 0.923 |
| SBSsi9 | 0.996 (0.030) | 0.884 |
| SBSsi10 | 1.076 (0.030) | 0.908 |
| SBSsi11 | 1.024 (0.030) | 0.908 |
| SBSsi12 | 0.994 (0.029) | 0.882 |

Note: SBS= sense of beloning; as= Social acceptance; si= sense of inclusion.

Table A3. Descriptive of the original pool of 16 items of GEQ scale.

| *Item* | *M* | *SD* | *Skew* | *Kurtosis* |
| --- | --- | --- | --- | --- |
| GEQso1 | 6.44 | 1.68 | -0.77 | 0.40 |
| GEQso2 | 6.49 | 1.66 | -0.78 | 0.40 |
| GEQso3 | 7.27 | 1.46 | -0.73 | -0.16 |
| GEQso4 | 6.86 | 1.69 | -0.60 | -0.32 |
| GEQt5 | 6.99 | 1.65 | -0.57 | -0.28 |
| GEQt6 | 6.88 | 1.65 | -0.57 | -0.28 |
| GEQt7 | 7.12 | 1.57 | -0.87 | 0.48 |
| GEQt8 | 7.01 | 1.57 | -0.72 | 0.08 |
| GEQsi9 | 7.51 | 1.61 | -1.22 | 1.21 |
| GEQsi10 | 7.37 | 1.58 | -1.22 | 1.21 |
| GEQsi11 | 7.24 | 1.70 | -1.36 | 1.75 |
| GEQsi12 | 7.50 | 1.60 | -1.35 | 1.50 |
| GEQti13 | 7.28 | 1.81 | -1.35 | 1.50 |
| GEQti14 | 7.22 | 1.78 | -1.22 | 1.05 |
| GEQti15 | 7.06 | 1.78 | -1.25 | 1.38 |
| GEQti16 | 7.05 | 1.74 | -1.05 | 0.76 |

Note: GEQ= Group Environment Questionnaire; so= Social Cohesion; t= Task Cohesion; si= Social Attraction to the Group; ti= Task Attraction to the Group

Table A4. Unstandardized Loadings (with standard errors) and Standardized Loadings of the entire pool of 16 items of GEQ scale included in the CFA.

| *Item* | *Unstandardized Loadings* | *Standardized Loadings* |
| --- | --- | --- |
| GEQso1 | 1.000 (-) | 0.881 |
| GEQso2 | 0.947 (0.030) | 0.835 |
| GEQso3 | 0.993 (0.031) | 0.875 |
| GEQso4 | 1.073 (0.030) | 0.945 |
| GEQt5 | 1.000 (-) | 0.896 |
| GEQt6 | 1.033 (0.026) | 0.888 |
| GEQt7 | 1.077 (0.029) | 0.926 |
| GEQt8 | 1.093 (0.028) | 0.939 |
| GEQsi9 | 1.000 (-) | 0.940 |
| GEQsi10 | 0.974 (0.019) | 0.916 |
| GEQsi11 | 0.977 (0.023) | 0.918 |
| GEQsi12 | 1.010 (0.019) | 0.949 |
| GEQti13 | 1.000 (-) | 0.956 |
| GEQti14 | 0.892 (0.033) | 0.853 |
| GEQti15 | 0.900 (0.036) | 0.861 |
| GEQti16 | 0.855 (0.033) | 0.818 |

Note: GEQ= Group Environment Questionnaire; so= Social Cohesion; t= Task Cohesion; si= Social Attraction to the Group; ti= Task Attraction to the Group
